# Universal broad-spectrum mucosal vaccine design for human coronaviruses inspired by artificial antibodies

**DOI:** 10.1038/s41541-026-01375-2

**Published:** 2026-01-28

**Authors:** Yan Wu, Jia Lu, Lijuan Fang, Xinlan Chen, Chenshu Zhao, Zhongfa Zhang, Xuerui Zhu, Xiao Gao, Haoyu Li, Yingrui Yan, Jian Shi, Jing Zhang, Pengfei Zhou, Xiaoyan Pan

**Affiliations:** 1 State Key Laboratory of Virology and Biosafety, Wuhan Institute of Virology, Chinese Academy of Sciences, Wuhan, China; 2 University of the Chinese Academy of Sciences, Beijing, China; 3 Wuhan YZY Biopharma Co., Ltd., Wuhan, China; 4 Hubei University, Wuhan, China

**Keywords:** Biotechnology, Immunology, Microbiology

## Abstract

Coronaviruses remain a challenge due to the limited or incomplete protection provided by existing vaccines, highlighting the need for improved antigen-based designs that can reduce mortality, block transmission, and provide long-lasting, broad-spectrum protection. In this study, we adapted artificial antibody strategies to display receptor-binding domains (RBDs) from representative human coronaviruses, utilizing an engineered human IgG1 framework modified at the Fab and Fc domains to support diverse antigen presentation and enhanced immunopotentiation. The results indicate that bivalent, tetravalent, and multivalent RBD constructs developed within this framework confer broad-spectrum immune protection against severe acute respiratory syndrome coronavirus 2 and other pathogenic coronaviruses. Moreover, Fc-mediated antigen delivery, primarily engaging the neonatal Fcγ receptor, enhances mucosal, cellular, and sustained immune responses. This underscores the versatility and practical utility of the modified IgG1 framework, based on artificial antibody strategies, for developing broad-spectrum mucosal vaccine antigens, representing promising vaccine candidates targeting human coronaviruses.

## Introduction

Severe acute respiratory syndrome coronavirus (SARS-CoV), Middle East respiratory syndrome coronavirus (MERS), and SARS-CoV-2 have fatality rates ranging from 3% to 40%^1^. Since early 2020, SARS-CoV-2 has given rise to five major variants of concern: Alpha, Beta, Gamma, Delta, and Omicron^2^. Among them, Omicron, first identified in December 2021, has further evolved into several sublineages, including BA.1, BA.1.1, BA.2, BA.3, BA.4/5, BQ.1, BF.7, XBB.1.5, XBB.1.16, EG.5, JN.1, and KP.3^3^ (https://www.who.int/activities/tracking-SARS-CoV-2-variants). With greater transmissibility but lower virulence than the original strain, Omicron has circulated for over three years and is now considered the fifth seasonal human CoV (HCoV), joining HCoV-OC43, HCoV-HKU1, HCoV-NL63, and HCoV-229E^4^. The recently identified HKU5 coronavirus may also pose risks^5^, highlighting the continued threat to humans. However, no broad-spectrum universal vaccines exist for these HCoVs, including SARS-CoV-2 and its variants.

HCoVs use the spike (S) protein’s receptor-binding domain (RBD) to bind host cell receptors, including human angiotensin-converting enzyme 2 (hACE2), dipeptide peptidase, sialic acid, and aminopeptidase, making the RBD a major vaccine target. However, frequent mutations in the SARS-CoV-2 RBD affect its transmissibility, virulence, and immunogenicity, complicating the efficacy of current monovalent vaccines targeting the RBD or the S protein^6^. While mutations contribute significantly to breakthrough infections, the lack of robust mucosal immunity in the upper respiratory tract also reduces the protection vaccines offer against airborne pathogens like HCoVs^7^. Thus, it is essential to develop vaccines that provide robust, broad-spectrum mucosal protection against HCoVs.

HCoV vaccine development faces two primary obstacles: rapid mutations within the RBD region that facilitate immune evasion and the ability of pathogens to infiltrate the respiratory mucosa, thereby circumventing systemic immunity. Broad-spectrum vaccine strategies—such as antigen mixtures^8^, intramolecular concatenation^9^, mosaic nanoparticles^10^, consensus sequences^11^, conserved proteins or epitopes^12,13^, and sequential vaccination^14^—have demonstrated broad-spectrum efficacy against SARS-CoV-2 in clinical trials and proof-of-concept studies. Nevertheless, the antigen mixture approach may lead to heterogeneous immune responses^15^, which may be due to intrinsic variations in antigen immunogenicity, differential antigen uptake, and antigen-presenting cells (APC) processing. Further challenges include conformational masking, inclusion of non-relevant antigens, limited protective breadth, manufacturing complexities, and incompletely understood mechanisms of action^16^.

Vaccines can trigger mucosal immunity by being delivered directly to the mucosal surfaces^17–19^, or by targeting the neonatal Fcγ receptor (FcRn) to remotely deliver antigens through immunoglobulin G (IgG) Fc domains or human serum albumin^20–22^. Notably, FcRn is widely found on mucosal epithelial cells and APCs, including dendritic cells and macrophages. Despite these strategies, most current methods do not provide comprehensive protection, flexibility, or safety, prompting researchers to combine different techniques to develop more effective vaccines^23^. It is worthing mentioning that, therapeutic antibodies based on IgG have been refined over decades and are now commonly used to treat cancers and autoimmune conditions^24,25^. Meanwhile, bispecific and multispecific antibodies have undergone clinical evaluation, demonstrating high safety and reliability.

This study leverages an artificial antibody strategy based on IgG1 to generate bivalent, trivalent, and tetravalent RBD antigens for HCoVs, with particular focus on SARS-CoV-2 and its variants. Unlike earlier versions of RBD dimers^26–28^, the design maintains and modifies the IgG1 Fc domain to enhance FcRn binding and prolong antigen retention, while reducing Fcγ receptor (FcγRs) binding to lower the risk of side effects, such as cytokine release syndrome (CRS). The binding capacity, immunogenicity, and protective efficacy of the antigens generated using this platform are assessed in vitro and in adult female BALB/c mice, hACE2-transgenic C57BL/6J mice, and Syrian hamsters. Findings show that this strategy offers broad, mucosal, and long-lasting protection in animal models, and provide insights regarding the associated immune mechanisms. Overall, this study introduces a universal antigen approach that could guide future respiratory vaccine development and aid in the design of candidate vaccines against pathogenic HCoVs.

## Results

### Modified framework enhances antigen presentation and mucosal barrier penetration via FcRn binding

**Fig. 1:**
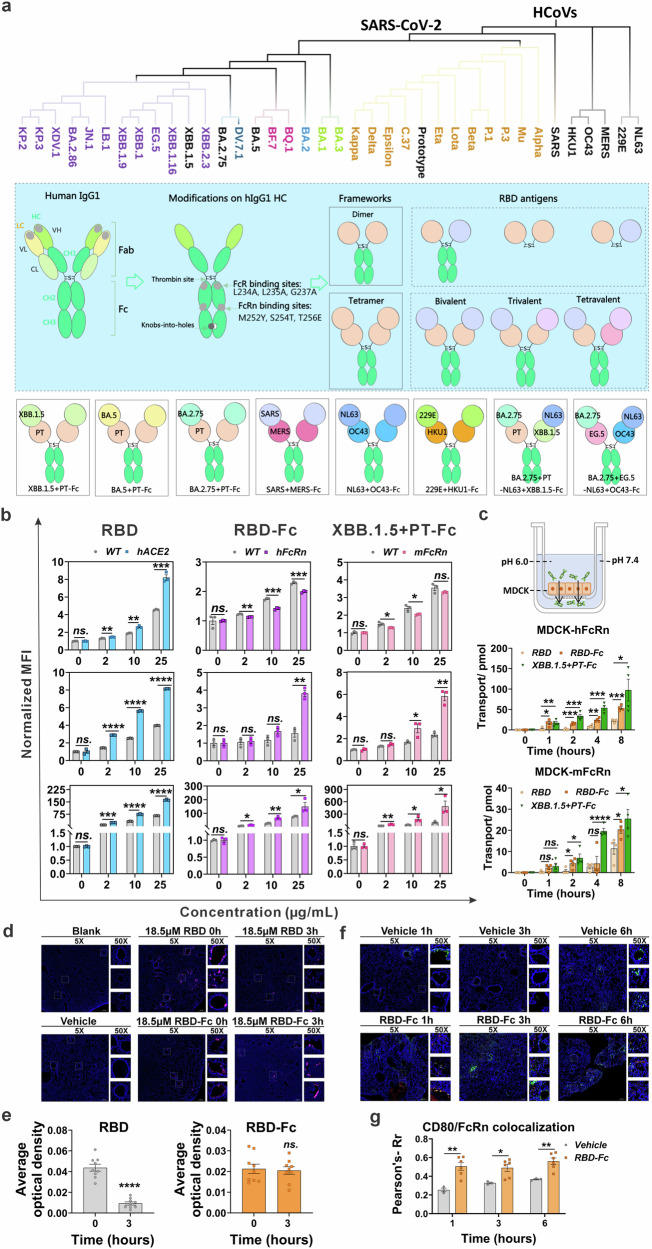
Rational design and characterization of the framework and antigens. **a** Schematic of the framework showing a modified IgG1 heavy chain after eliminating FcγR (containing C1q) binding and improving FcRn binding, enabling mono-, bi-, tri-, and tetravalent antigen production. Phylogenetic analysis of SARS-CoV-2 and other HCoV spike proteins, with six bivalent and two tetravalent antigens was successfully produced, and illustrated with iTOL (https://itol.embl.de/). **b** Binding of the bare RBD dimer, RBD-Fc, and XBB.1.5 + PT-Fc to hACE2, mouse, and human FcRn assessed by flow cytometry in overexpressing vs. wild-type cell lines. **c** Transwell transcytosis model with MDCK cells overexpressing mouse or human FcRn; cells with bare RBD dimer, RBD-Fc, or XBB.1.5 + PT-Fc on the lateral side were collected at 1, 2, 4, and 8 h, and their molar concentrations determined by ELISA. **d** Fluorescently labeled antigens (fuchsia) with equal moles (18.5 μM, bare RBD, 50 μg; RBD-Fc, 100 μg) were administered intranasally to adult BALB/c mice (*n* = 3), with bare RBD dimer as a control. The lungs were dissected at various time points to examine the location of antigens and the activation of antigen-presenting cells. Three representative images (50×) of the bronchus pulmonalis are shown with a panoramic view of the lungs (5×). DAPI staining (blue) was employed to indicate the position of the nucleus. **e** Antigen retention near bronchial pulmonalis quantified via fluorescence. **f** CD80 (green) and FcRn (red) double-positive antigen-presenting cells in the submucosa were imaged (50×) and mapped along a panoramic lung view (5×). DAPI staining (blue) was employed to indicate the position of the nucleus. **g** Co-localization of CD80 and FcRn statistically analyzed for multiple sections. Unpaired *t*-tests with the Mann–Whitney U-tests, ns*: p* > 0.05, *p < 0.05, **p < 0.01, ***p < 0.001, ****p < 0.0001.

A novel antigen framework was developed based on human IgG1 to optimize antigen presentation and the ability to cross mucosal barriers. In this design, the light chain was removed, and the heavy chain was specifically modified: mutations (L234A, L235A, and G237A)^29,30^ were introduced to attenuate classical FcγR binding, thereby reducing the risk of side effects, including CRS; other mutations (M252Y, S254T, and T256E)^31^ enhanced binding to FcRn, effectively extending antigen half-life and improving penetration of epithelial mucosa^31^. To further improve structural flexibility and production efficiency, features such as thrombin restriction site and a knobs-in-holes element were incorporated, facilitating the successful generation of mono-, bi-, tri-, tetravalent, and non-Fc antigens. Using this framework, RBDs from various HCoVs, including two highly pathogenic viruses (SARS and MERS), four seasonal HCoVs (NL63, OC43, 229E, and HKU1), and multiple SARS-CoV-2 variants, which are analyzed based on their evolutionary relationships and serotype classification^32^, were prepared through this framework, intending to cover as many HCoVs as possible (Fig. 1a, Fig. S1).

Representative bivalent antigens, such as XBB.1.5 + PT-Fc, were selected for further analysis. The binding affinity of XBB.1.5 + PT-Fc to ACE2 and FcRn was evaluated using fluorescently labeled constructs. These antigens—including XBB.1.5 + PT-Fc, PT RBD-Fc, and the non-Fc PT RBD dimer—were incubated with cell lines stably expressing human ACE2 (hACE2), human FcRn (hFcRn), or mouse FcRn (mFcRn), with wild-type (WT) cell lines serving as controls. Flow cytometry results showed that all three antigens bound robustly to hACE2-overexpressing HEK293T cells. Additionally, XBB.1.5 + PT-Fc and RBD-Fc demonstrated increased binding to MDCK cells overexpressing hFcRn and mFcRn, whereas the non-Fc RBD dimer lacked this capacity (Fig. 1b). These findings confirm that the engineered antigens retain the ability to bind ACE2 through the RBD and to bind FcRn via the Fc region, preserving their active conformations.

Transwell assays were conducted to assess mucosal penetration. Both XBB.1.5 + PT-Fc and RBD-Fc efficiently crossed MDCK cells overexpressing hFcRn or mFcRn, whereas the bare RBD dimer did not, demonstrating Fc-mediated, time-dependent transcytosis (Fig. 1c). In vivo studies that mice were administrated with equal mole antigens, further revealed that fluorescently labeled RBD-Fc persisted in the lung mucosa for at least 3 h, while the bare RBD dimer was rapidly eliminated within 3 h, likely due to physical, chemical or immune processes in the lung mucosal environment (Fig. 1d, e). This suggests that the Fc modification significantly prolongs the antigen’s presence in the mucosa.

Moreover, RBD-Fc seemingly activated or recruited APCs to the submucosa, as evidenced by markedly increased CD80 expression and colocalization with FcRn (Fig. 1f, g). Collectively, these results demonstrate that this modified framework can generate antigens that enhance presentation and immune activation, supporting the advancement of respiratory or mucosal vaccines.

### Fc domain potentiates humoral and cellular immunity, particularly via intranasal administration

**Fig. 2:**
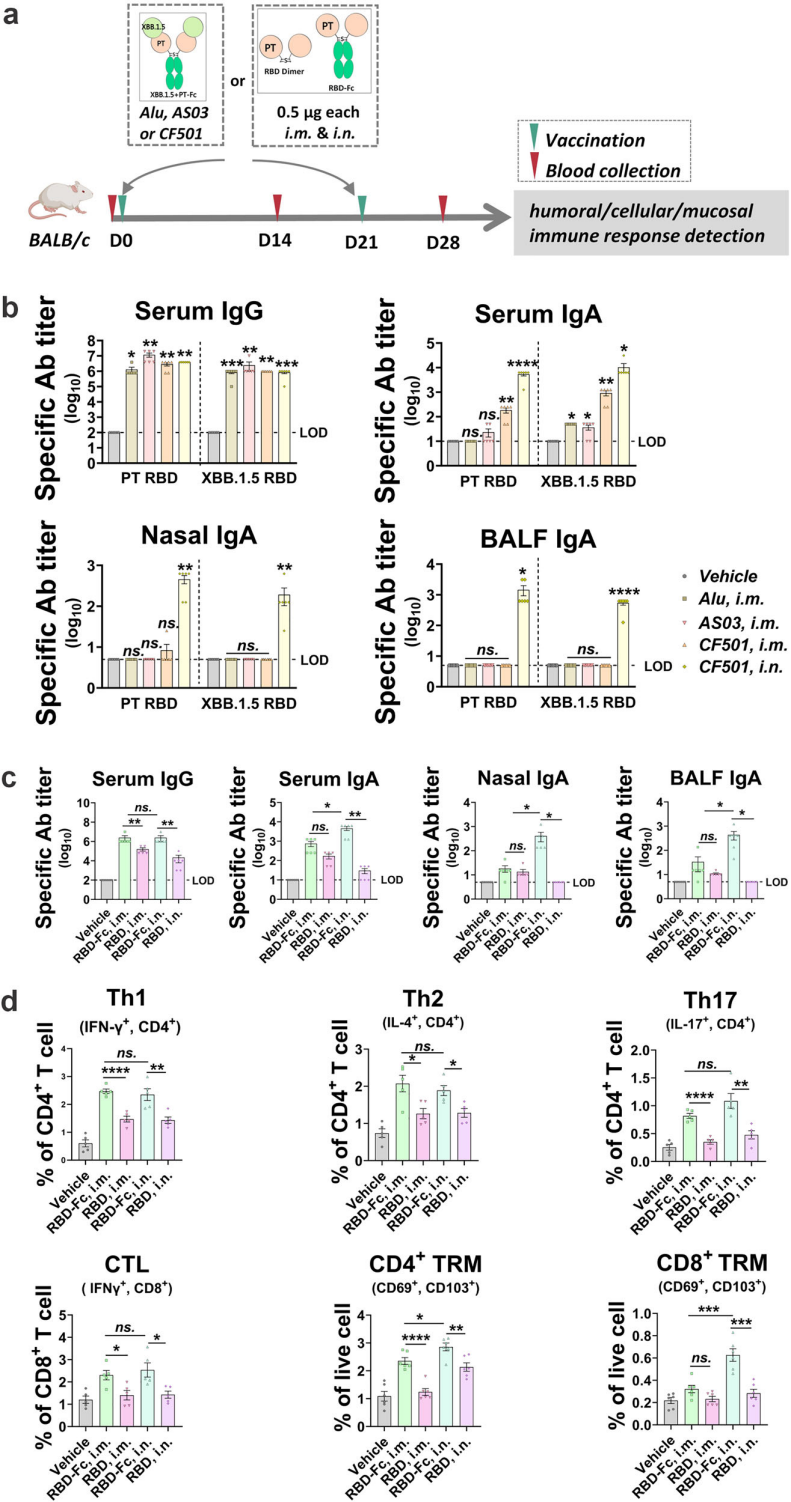
Modified Fc induces strong T cell and mucosal immune responses. **a** Adult female BALB/c mice (*n* = 6) were vaccinated with 1 µg of XBB.1.5 + PT-Fc antigen, adjuvanted with Alu, AS03, or CF501, and administered intramuscularly (i.m.) or intranasally (i.n.); or BALB/c mice (*n* = 5–6) were vaccinated with 1 μg of RBD-Fc and an equal molar of bare RBD dimer (~0.5 μg) via i.m. and i.n. Serum samples were collected biweekly, and tissues were collected for analysis. **b**, **c** RBD (PT or XBB.1.5)-specific IgG and IgA in serum, nasal fluid, and bronchoalveolar lavage fluid (BALF) measured by ELISA. The limit of detection (LOD) was established as follows: 100 for serum IgG, 10 for serum IgA, 5 for nasal and BALF IgA. **d** Th1/2/17 CD4^+^ T cells, CD8^+^ T cells, cytotoxic T lymphocytes (CTLs), and tissue-resident memory (TRM) CD4^+^ and CD8^+^ T cells were determined via flow cytometry after ex vivo restimulation with peptide pools, and analyzed by FlowJo 10. Unpaired *t*-tests with the Mann–Whitney U-tests, ns: *p* > 0.05, *p < 0.05, **p < 0.01, ***p < 0.001, ****p < 0.0001.

Antigens and the framework were then subjected to immune immunopotentiation investigation. Mice were inoculated with antigens via either intramuscular (i.m.) injection or intranasal (i.n.) inhalation, following a two-dose regimen with a 3-week interval. Serum samples were collected at two-week intervals to assess humoral immune responses, and spleens were harvested one week after the second dose for cellular immune response analysis (Fig. 2a).

The representative antigen, XBB.1.5 + PT-Fc, was formulated with classical muscular adjuvants—aluminum hydroxide (Alu) and AS03—as well as the novel stimulator of interferon gene (STING) agonist-based candidate adjuvant, CF501^33^, to evaluate optimal formulation strategies. Administration of XBB.1.5 + PT-Fc with Alu, AS03, or CF501 resulted in robust serum-specific IgG titers (1:10^6^ to 1:10^7^) against the prototype (PT) and XBB.1.5 RBD, regardless of i.m. and i.n. vaccination route. Notably, i.n. delivery produced higher serum, nasal, and alveolar-specific IgA levels compared to i.m. administration (Fig. 2b). Accordingly, XBB.1.5 + PT-Fc adjuvanted with CF501 and delivered intranasally generated high titers of specific antibodies in serum and mucosal compartments.

To further characterize the immune response driven by the Fc region, RBD-Fc was administered with CF501 at a molar mass equal to that of bare RBD dimer (control) via both i.m. and i.n. routes. When adjuvanted with CF501, i.m. delivery of RBD-Fc induced greater IgG production than the bare RBD dimer, although IgA levels did not differ significantly between the two groups. In contrast, i.n. administration of RBD-Fc resulted in increased systemic and mucosal (respiratory) IgA and IgG levels (Fig. 2c), indicating that Fc enhanced immune activity across multiple compartments. Similar trends were observed when comparing i.n. administration of BA.5 + PT-Fc and its non-Fc (bare) counterpart (Fig. S2).

Regarding cellular immunity, RBD-Fc administration led to increased populations of Th1, Th2, Th17, cytotoxic CD8^+^ T lymphocytes (CTLs), and tissue-resident memory (TRM) CD4^+^ and CD8^+^ T cells following either i.m. or i.n. vaccination, relative to bare RBD dimer. Particularly, i.n. delivery of RBD-Fc significantly increased the proportion of Th17 and TRM CD4^+^ and CD8^+^ T cells (Fig. 2d), key markers of enhanced mucosal immunity.

Together, these findings demonstrate that the engineered antigen framework effectively induces robust systemic and mucosal humoral and cellular immune responses, with the intranasal route providing especially pronounced benefits.

### Bivalent antigen provides broad-spectrum protection against SARS-CoV-2 at low doses

**Fig. 3:**
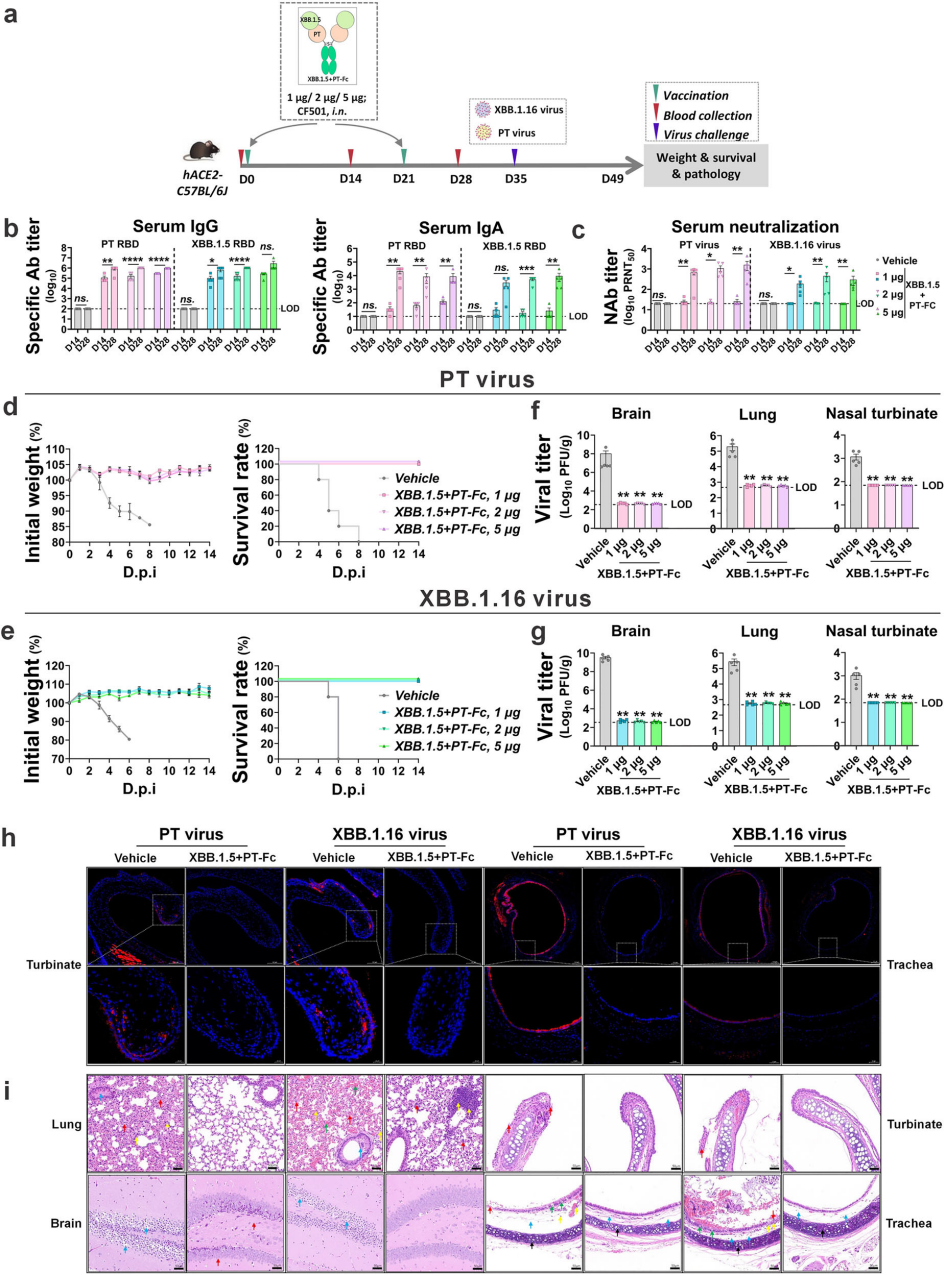
Low-dose bivalent antigens provide complete protection in a lethal challenge model. **a** Experimental design: Adult female hACE2-transgenic C57BL/6J mice (*n* = 5) were vaccinated with 1, 2, or 5 μg of XBB; 1.5 + PT-Fc antigen adjuvanted with CF501, via i.n. Two weeks after the second dose, mice were challenged intranasally with a lethal dose of SARS-CoV-2 (PT or XBB.16). The brain, lung, and turbinate tissues were collected for viral and pathological analyses. **b** Post-vaccination sera were tested for RBD (PT or XBB.1.5) cross-reactive IgG and IgA by ELISA. The limit of detection (LOD) for serum IgG and IgA was 100 and 10, respectively. **c** The 50% plaque reduction neutralization titer (PRNT_50_) was assessed against authentic SARS-CoV-2 variants, including PT and XBB.1.16, using sera from each mouse; the LOD was 20. **d**, **e** Body weight and survival curves plotted based on daily data, using the vehicle group as a control. **f**, **g** Viral loads in the brain, lungs, and turbinates collected at the experimental endpoint were detected by the plaque method, and the LOD was 350 PFU/g (brain), 466 PFU/g (lungs), and 70 PFU/g (nasal turbinate). **h** Representative immunofluorescence images of viral antigens (N protein, red) in the turbinates and trachea; nuclei are stained with DAPI (blue). **i** Representative hematoxylin and eosin staining images; colored arrows indicate obvious pathological features: (i) lungs: red indicates alveolar epithelial cells proliferated, alveolar atrophy, and alveolar walls thickened; green denotes congested and dilated capillaries within the alveolar wall; blue indicates protein mucus in the bronchus lumen; yellow denotes inflammatory cell infiltration; (ii) brain: red indicates neuronal cell degeneration, karyopyknosis, and basophilia; blue denotes neuronal cell edema; (iii) turbinate: red indicates mucosal layer epithelial cells arranged irregularly with cellular shedding; (iv) trachea: red indicates epithelial cell shedding of the mucosal layer; green denotes mild edema of epithelial cells (i.e., cellular swelling and pale cytoplasm); blue indicates mild edema in the submucosal layer with enlarged tissue spaces; yellow represents inflammatory cell infiltration; black indicates hyaline cartilage. Unpaired *t*-tests with the Mann–Whitney *U* tests, ns: *p* > 0.05, *p < 0.05, **p < 0.01, ***p < 0.001, ****p < 0.0001.

In-depth studies were conducted to evaluate the protective efficacy of the representative bivalent antigen, XBB.1.5 + PT-Fc. Utilizing a two-shot regimen, XBB.1.5 + PT-Fc adjuvanted with CF501 was administered intranasally to hACE2-transgenic C57BL/6J mice. The approach enabled precise assessment of in vivo protection (Fig. 3a).

Antibody analyses revealed that XBB.1.5 + PT-Fc elicited robust IgG (1:10^6^) and IgA (1:10^4^) titers against both variant RBDs, at doses ranging from 1 to 5 µg. Notably, an increase in IgA titers was observed following the second dose (Fig. 3b). Serum neutralization assays further demonstrated enhanced neutralization activity against PT and XBB.1.16 (E180V and K478R from XBB.1.5) viruses following the second dose, even at the lowest tested dose of 1 μg (Fig. 3c).

Upon challenge with PT or XBB.1.16 viruses, control vehicle mice receiving only adjuvant experienced rapid weight loss beginning two days post-infection (dpi) and died between 4 and 8 dpi. In contrast, mice immunized with XBB.1.5 + PT-Fc maintained stable body weights and achieved 100% survival following lethal challenge (Fig. 3d, e). Endpoint analyses of brain, lung, and turbinate tissues confirmed the absence of live virus in the XBB.1.5 + PT-Fc groups (Figs. 3f, g). Histologic immunofluorescence staining of turbinate and trachea sections further validated the lack of residual virus in the upper respiratory tract (Fig. 3h).

Histopathological examination demonstrated that vaccination with XBB.1.5 + PT-Fc markedly reduced damage caused by PT or XBB.1.16 virus in the brain, lungs, turbinates, and trachea. Additionally, the vaccine alleviated pathological manifestations, including tissue damage, structural abnormalities, fluid leakage, and inflammatory infiltration. Only symptoms that are challenging to resolve within a short period persisted (Fig. 3i).

**Fig. 4:**
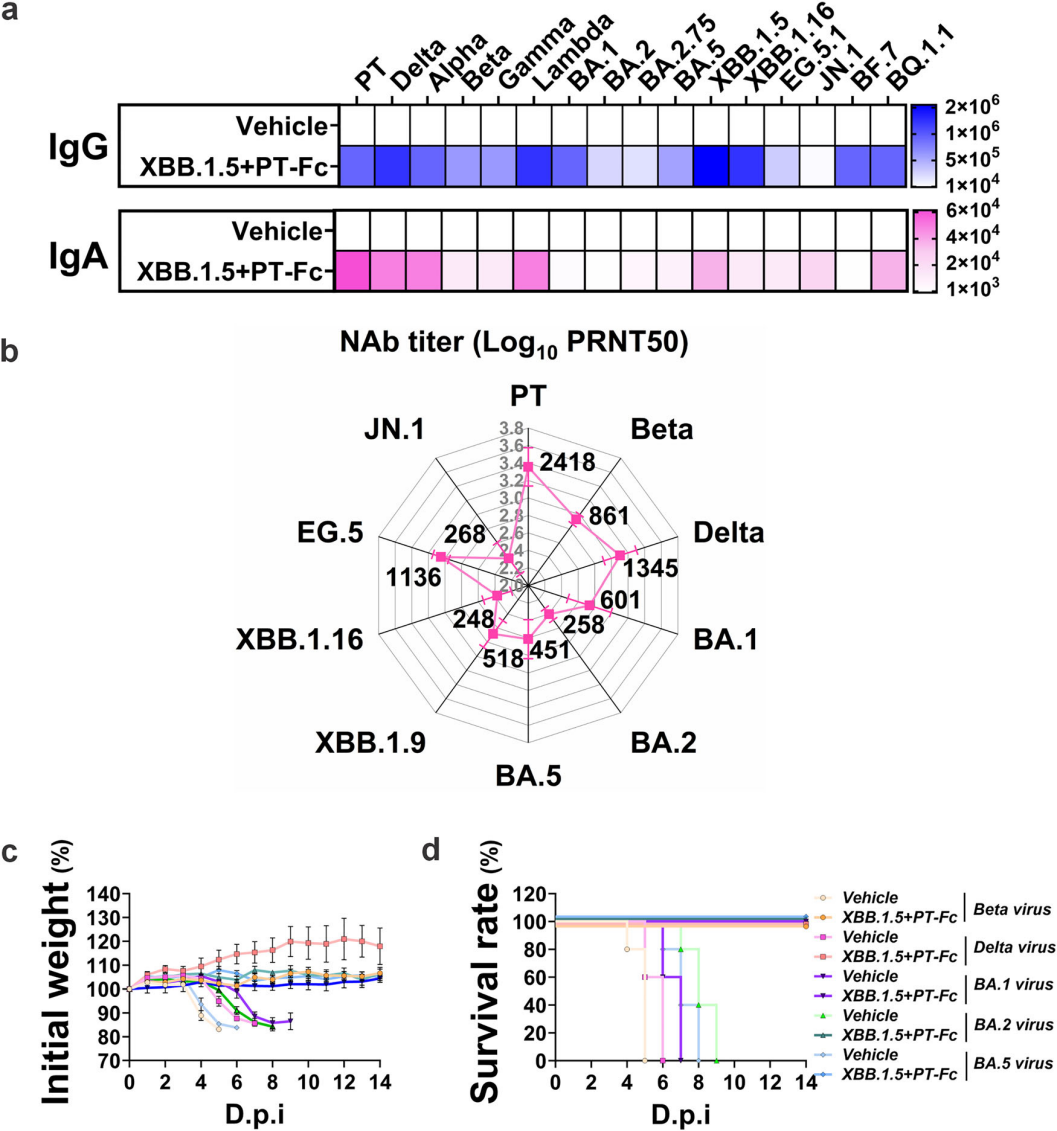
Bivalent antigen confers broad-spectrum protection against SARS-CoV-2 and its variants. **a** Adult hACE2-transgenic C57BL/6J mice (*n* = 5) received a two-dose regimen of XBB.1.5 + PT-Fc (1 µg), followed by serum sampling and subsequent lethal viral challenge. Cross-binding IgG and IgA induced by the bivalent antigen (XBB.1.5 + PT-Fc) were detected by ELISA against the RBDs of emerging SARS-CoV-2 variants of concern. The vehicle group serves as a control. **b** Cross-neutralization antibodies elicited by the bivalent antigen (XBB.1.5 + PT-Fc) evaluated by the plaque reduction neutralization test (PRNT), and geometric mean titers, calculated from PRNT_50_ values, are presented. **c** Mice were challenged with a panel of SARS-CoV-2 variants (Beta, Delta, BA.1, BA.2, and BA.5) two weeks after their second dose. Body weight measurements were recorded daily. **d** Survival rates of challenged mice, with the vehicle group as the control.

The broad-spectrum protection conferred by XBB.1.5 + PT-Fc was further assessed against key SARS-CoV-2 variants. After the second dose, sera exhibited strong cross-binding and neutralizing activity, with detectable IgG and IgA responses for nearly all major variants, including JN.1, BF.7, and BQ.1.1 (Fig. 4a). Neutralization assays confirmed efficacy across PT and multiple SARS-CoV-2 lineages, including Beta, Delta, and Omicron subvariants BA.1, BA.2, BA.5, and BA.2 sublineages XBB.1.9, XBB.1.16, and JN.1, which cover the six serotypes of SARS-CoV-2 reported by the previous study^32^ (Fig. 4b). Mouse models immunized with XBB.1.5 + PT-Fc demonstrated complete protection against Beta, Delta, BA.1, BA.2, and BA.5 challenges, as well as PT and XBB.1.16 in Fig. 3, which are representatives from all six serotypes. In contrast, control vehicle mice exhibited sustained weight loss and mortality between 4 and 9 dpi (Fig. 4c, d). This demonstrated a wide protection spectrum is achieved by combining RBD antigens with a great evolutionary distance.

Additional bivalent antigens—including BA.5 + PT-Fc and BA.2.75 + PT-Fc—were prepared, administered, and evaluated using the same protocols as XBB.1.5 + PT-Fc. Measurements of neutralizing antibody responses, body weight, survival rates, and viral loads demonstrated strong antigen-specific immunity and complete protection (Fig. S3).

These results highlight that bivalent antigens developed through this platform—such as XBB.1.5 + PT-Fc—provide broad-spectrum protection against SARS-CoV-2 variants. Moreover, the bivalent strategy can be adapted to address various variant combinations and effectively combat emerging variants.

### Bivalent antigen blocks SARS-CoV-2 airborne transmission through the hamster respiratory tract

**Fig. 5:**
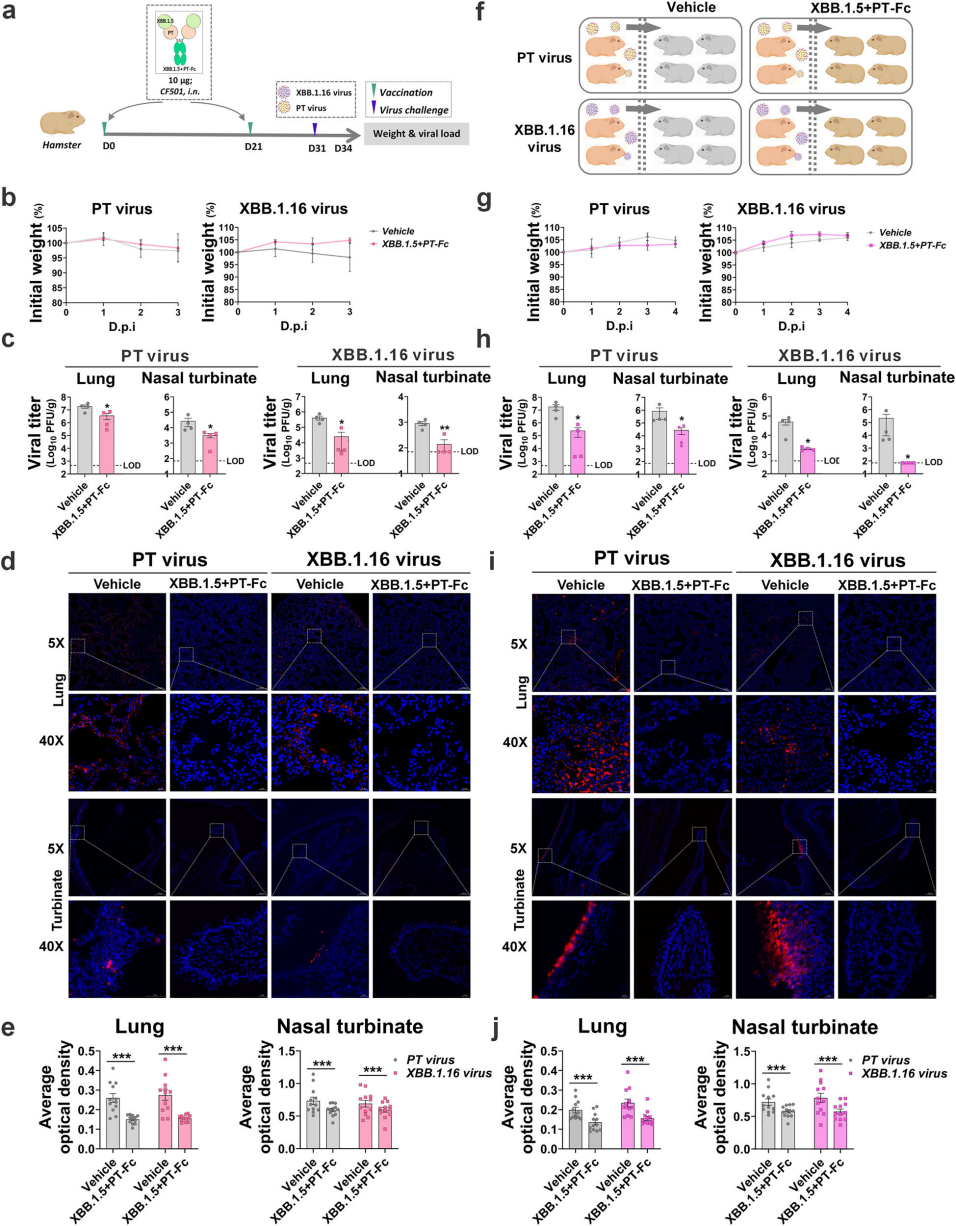
Bivalent antigen blocks infection and transmission in a hamster model. **a**, **f** Adult Syrian golden hamsters (*n* = 4) were vaccinated with 10 µg of XBB.1.5 + PT-Fc antigen adjuvanted with CF501, via intranasal (i.n.) route. Vaccination followed a two-dose schedule; ten days after the second dose, animals were challenged with PT or XBB.1.16 via i.n. administration or exposed to a co-housing airborne transmission model. Viral loads in respiratory tissues, including the lungs and turbinates, were assessed three days post-exposure or co-housing. **b**, **g** Daily recorded body weights. **c**, **h** Viral loads in the lungs (limit of detection [LOD] was 466 PFU/g) and turbinates (LOD was 70 PFU/g) following challenge with PT or XBB.1.16. **d**, **i** Immunofluorescence staining of viruses in the lungs and turbinates against the SARS-CoV-2 N protein (red) with DAPI nuclear staining (blue). Representative images from each group are shown in panoramic (5×) and local (40×) views. **e**, **j** Fluorescence signals from multiple views for each hamster. Unpaired *t*-tests with Mann–Whitney U-test, *p < 0.05, **p < 0.01, ***p < 0.001.

To evaluate the transmission-blocking capability of the bivalent antigen XBB.1.5 + PT-Fc, hamsters were immunized using the established mouse protocol and subsequently challenged intranasally with PT or XBB.1.16 virus (Fig. 5a). Since SARS-CoV-2 infection resulted in minimal impact on hamster body weight (Fig. 5b) and only minor pathological changes in the respiratory tract, viral load was selected as the primary assessment criterion. Hamsters vaccinated with XBB.1.5 + PT-Fc exhibited a marked reduction in viral loads within the lungs and nasal turbinates after challenge with PT or XBB.1.16 (Fig. 5c). These findings were corroborated by representative images of lung and turbinate images (Fig. 5d) and by quantitative fluorescence analyses (Fig. 5e), which demonstrated significantly diminished viral load.

Further investigation utilized a co-housed airborne transmission hamster model^34,35^ to confirm the efficacy of XBB.1.5 + PT-Fc in blocking viral transmission and providing mucosal protection. In this model, vaccinated hamsters were housed with infected donors for 24 h (Fig. 5f). The vaccinated animals exhibited minimal weight changes (Fig. 5g) and significantly lower viral loads in the lungs and turbinates (Fig. 5h), irrespective of exposure to PT or XBB.1.5 + PT-Fc. Fluorescence imaging and quantitative analysis of respiratory tissues revealed fewer viruses in the upper (turbinate) and lower (lung) respiratory tract (Fig. 5i, j). Collectively, these results verify that the bivalent antigen XBB.1.5 + PT-Fc can broadly prevent airborne SARS-CoV-2 transmission and confer comprehensive respiratory immunity in a hamster model.

### One-year immune protection conferred by the bivalent antigen

**Fig. 6:**
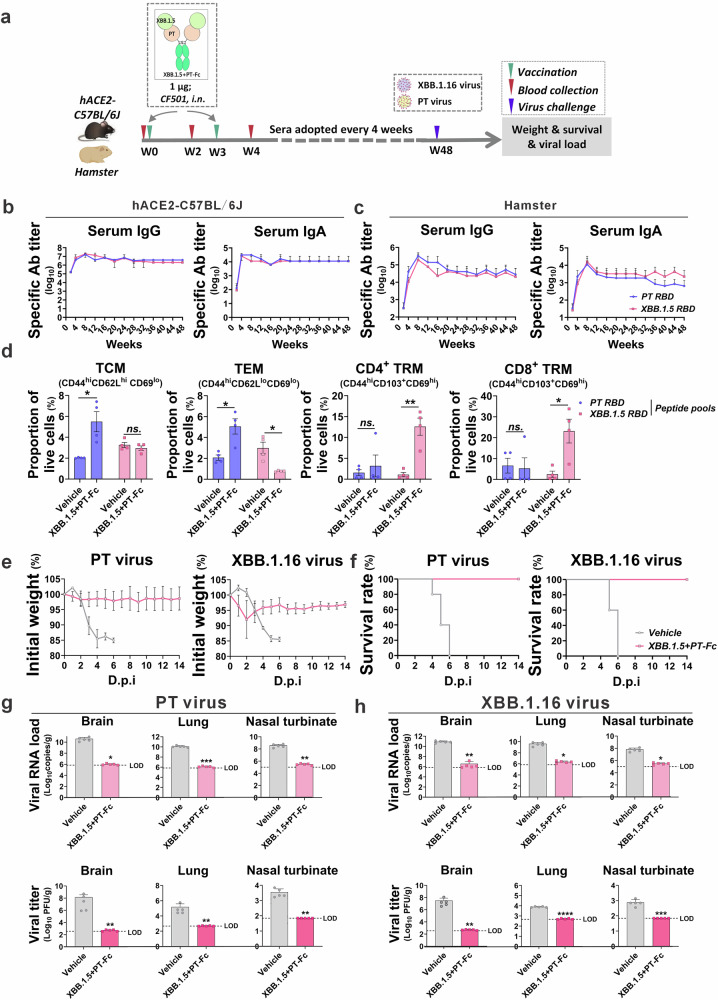
Bivalent antigen provides protection for at least 48 weeks. **a** Adult hACE2-transgenic C57BL/6J mice (*n* = 5) or Syrian golden hamsters (*n* = 4) received two intranasal doses of 1 µg of XBB.1.5 + PT-Fc antigen adjuvanted with CF501, three weeks apart. Serum samples were collected every 4 weeks and subjected to a lethal challenge. Sera from mice (**b**) or hamsters (**c**) were assessed for PT or XBB.1.5 RBD-specific IgA or IgG. **d** Memory T cell analysis upon ex vivo restimulation with peptide pools from PT or XBB.1.5 RBD. Body weight changes (**e**) and survival rates (**f**) of mice after lethal challenge with PT or XBB.1.16 virus. **g**, **h** Viral copies (upper panel) and viral titers (lower panel) in the brain, lungs, and turbinates at the experimental endpoint. The limit of detection (LOD) for viral copies was established as follows: brain and lung, 7 × 10^5^ copies/g, nasal turbinate, 1 × 10^5^ copies/g. LOD for viral titers: 350 PFU/g (brain), 466 PFU/g (lungs), and 70 PFU/g (nasal turbinate). Unpaired *t*-tests with Mann–Whitney U-tests, ns: *p* > 0.05, *p < 0.05, **p < 0.01, ***p < 0.001, ****p < 0.0001.

To evaluate the duration of immunity conferred by bivalent antigens, hACE2-transgenic C57BL/6J mice and hamsters were vaccinated and monitored for 48 weeks. Serum samples were collected at 4-week intervals, enabling the assessment of specific antibody titers and immune responses to lethal viral challenges (Fig. 6a). Throughout the study, mice maintained consistently high serum IgG (1:10^6^) and IgA (1:10^4^) titers specific to PT or XBB.1.5 RBD, with no significant decline observed (Fig. 6b). In hamsters, serum IgG (1:10^5^) and IgA (1:10^4^) titers showed a moderate decrease beginning at week 8, but levels remained relatively high through the 48th week (Fig. 6c). Flow cytometry identified sustained central memory T cell and effector memory T cell (TEM) populations targeting PT RBD as well as TRM CD4+ and CD8 + T cells reactive to XBB.1.5 RBD, for nearly one year (Fig. 6d). These findings suggest that the bivalent antigen strategy promotes a durable immune response with potential in vivo protective effects.

Upon challenge with a lethal dose of PT or XBB.1.16 virus, vehicle-treated mice experienced rapid weight loss and died within 4–6 dpi. In contrast, all mice vaccinated with XBB.1.5 + PT-Fc survived, with only minor and transient weight loss observed in a few individuals (Fig. 6e, f). Viral load analysis in the brain, lungs, and turbinates indicated complete viral clearance, as evidenced by the absence of detectable viral gene copies and live virus titers (Fig. 6g, h).

Collectively, these results demonstrate that the bivalent antigen approach induces robust and long-lasting immunity, offering protective efficacy that extends beyond broad-spectrum mucosal defense.

### Mixed bivalent antigens induce pan cross-binding antibodies against HCoVs

**Fig. 7:**
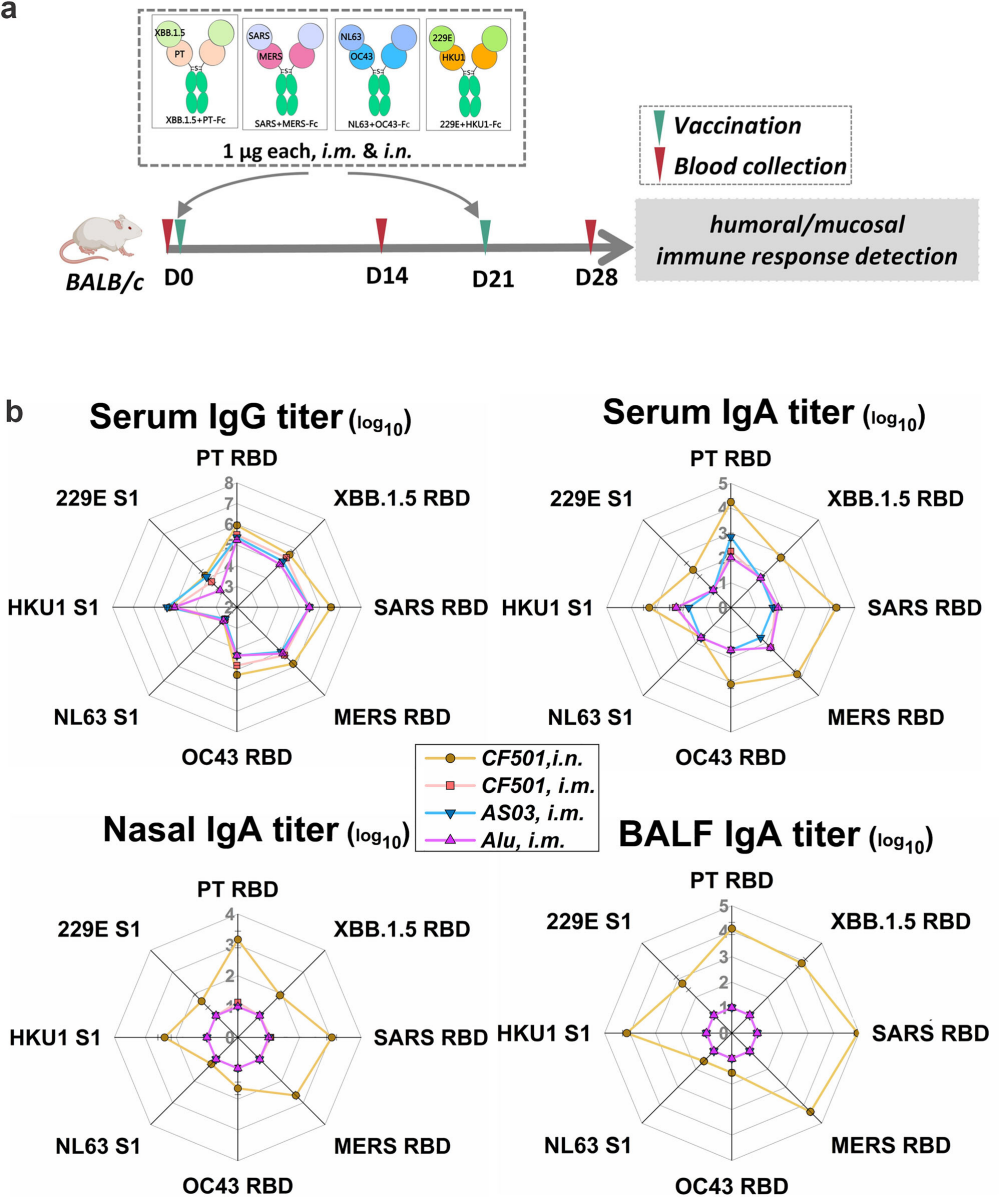
Mixture of bivalent antigens triggers pan-immune responses. **a** Adult BALB/c mice (*n* = 5) were vaccinated with four bivalent antigens, each including RBDs or spike subunit 1 (S1) from SARS-CoV-2 (XBB.1.5 and PT), SARS-CoV, MERS-CoV, and HCoV (OC43, NL63, HKU1, and 229E), 1 µg per antigen, with Alu, AS03, or CF501 adjuvants via intramuscular (i.m.) or intranasal (i.n.) routes. After two doses, serum, nasal lavage fluid, and bronchoalveolar lavage fluid (BALF) were collected. **b** IgG and IgA levels in sera, and IgA levels in nasal fluid and BALF against the indicated RBD or S1.

Given the superior protection afforded by XBB.1.5 + PT-Fc against SARS-CoV-2, it was combined with bivalent antigens from major HCoVs—SARS, MERS, OC43, NL63, 229E, and HKU1—to further broaden the protection scope, which would cover almost all HCoVs. Four bivalent antigens (1 µg each) were mixed and formulated with adjuvants Alu, AS03, or CF501 before being administered via i.n. or i.m. routes. Following the second dose, antibody levels were measured in serum, nasal, and alveolar lavage fluids (Fig. 7a). Mice receiving the antigen mixture with any adjuvant developed high RBD or S1-specific IgG titers (1:10^5^ to 1:10^6^) for PT, XBB.1.5, SARS, MERS, OC43, and HKU1. Notably, NL63 and 229E exhibited weaker responses across all adjuvant groups, likely reflecting their intrinsic antigenicity. CF501-adjuvanted i.n. vaccination significantly increased IgA levels in the serum, nasal cavity, and alveoli (Fig. 7b), supporting the potential for pan-HCoV protection using this bivalent antigen mixture.

### Broad-spectrum immunity achieved with tetravalent antigens

**Fig. 8:**
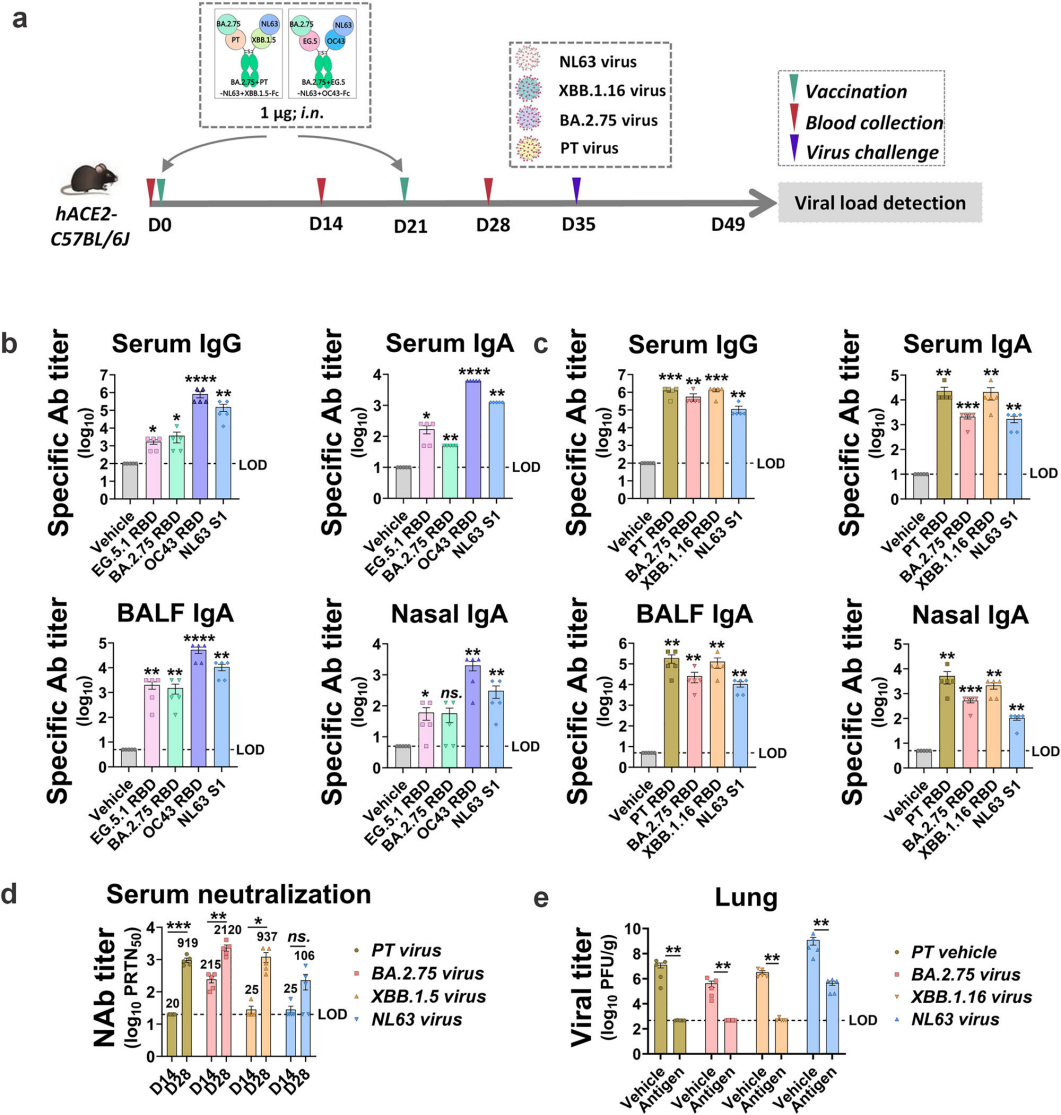
Tetravalent antigens provide cross-species protection. **a** Adult hACE2-transgenic C57BL/6 J mice (*n* = 5) vaccinated with 1 μg of tetravalent antigen (BA.2.75 + EG.5- NL63 + OC43-Fc or BA.2.75 + PT- NL63 + XBB.1.5-Fc) were analyzed. BA.2.75 + PT- NL63 + XBB.1.5-Fc-vaccinated mice were challenged with the four corresponding HCoVs: SARS-CoV-2 (PT, BA.2.75, and XBB.1.16) and HCoV NL63; lung viral loads were measured three days post-infection. **b** IgG or IgA in the serum, nasal fluid, and BALF against the RBD or S1 of SARS-CoV-2 (EG.5.1 and BA.2.75) and HCoVs (OC43 and NL63). The limit of detection (LOD) was established as follows: 100 (serum IgG), 10 (serum IgA), and 5 (nasal and BALF IgA). **c** IgG and IgA in the serum, nasal fluid, and BALF against SARS-CoV-2 (PT, BA.2, and XBB.1.16), RBD, and HCoV NL63 S1. The LOD for each item was the same as noted above. **d** PRNT_50_ against SARS-CoV-2 (PT, BA.2, and XBB.1.16) and HCoV NL63 in each serum sample; geometric mean titers calculated from the PRNT_50_ are shown. The LOD was 20. **e** Viral loads in the lungs quantified by the plaque method, the LOD was 466 PFU/g. Unpaired *t*-tests with Mann–Whitney U-tests, ns: *p* > 0.05, *p < 0.05, **p < 0.01, ***p < 0.001, ****p < 0.0001.

To broaden the scope of protection, a multispecific antibody strategy was used to generate tetravalent antigens. Mice were vaccinated with tetravalent antigens (BA.2.75 + EG.5-NL63 + OC43-Fc, or BA.2.75 + PT-NL63 + XBB.1.5-Fc) using the same protocol, and sera were analyzed after the second dose (Fig. 8a). Both antigen groups induced specific IgG responses to their respective RBDs or S1—EG.5.1, BA.2.75, OC43, and NL63, or PT, BA.2.75, XBB.1.16, and NL63. Similar trends were observed for IgA levels in the serum, nasal cavity, and alveoli (Fig. 8b, c). For the BA.2.75 + PT-NL63 + XBB.1.5-Fc group, neutralization assays and challenge experiments revealed high neutralizing antibody titers (Fig. 8d) and a significant reduction in lung viral load against PT, BA.2.75, XBB.1.16, and NL63 after the second dose (Fig. 8e). These findings confirm that tetravalent antigens provide broad-spectrum protection against diverse representative HCoVs, supporting the feasibility and effectiveness of this vaccination framework.

## Discussion

This study presents an intramolecular concatenation approach inspired by artificial antibody structures. Unlike previous schemes that combine two or three antigens in tandem^9,36^ or use polymeric elements to generate trimers^37^, our strategy leverages multivalent antibodies to present four proteins within a single molecule^38^. This design minimizes the obscuration often caused by direct tandem arrangements and reduces immune variability associated with mixtures of multiple molecules. The constructed antigens bind effectively to ACE2 and FcRn receptors, preserving the functional integrity of the RBD and Fc region—a result potentially attributed to the stable IgG1 heavy chain, which supports RBD extension.

This study employed a modified Fc region to enable safe penetration of mucosal barriers and efficient presentation of antigens to APCs, providing comprehensive immune protection. The unique environment and immune tolerance of the respiratory mucosa pose significant challenges for vaccine and adjuvant development^39^. Ensuring safety and efficacy is paramount, as damage to the respiratory mucosa can lead to inflammation, allergies, or fibrosis in the nasal cavity, bronchi, or lungs. The respiratory mucosal barrier comprises surface-attached mucus, tightly adherent epithelial cells, and a local immune environment within the lamina propria, which serves as the first line of defense against respiratory viruses^40^. Secretory IgA and resident CD4/8^+^ T cells are crucial for mucosal defense^7,41^ and require in situ induction, a feature not provided by current intramuscular vaccines.

Most respiratory virus vaccines currently in use or under development are viral vectored or mRNA-based^17,19,42^, but their long-term side effects, such as potential overactivation of innate immunity, remain underexplored^43^. Given the established safety and suitability of recombinant proteins for respiratory application, this approach was selected for the current study. Mucosal delivery was further improved by modifying the Fc portion of human IgG1 to facilitate tissue penetration via FcRn-mediated transcytosis at low pH, which extends the antigen’s half-life. The high expression of FcRn on mucosal epithelial cells and APCs supports rapid Fc adsorption, capture, and presentation in the slightly acidic mucosa^44^, thereby enhancing immune responses. In vitro and in vivo studies confirmed improved antigen retention and increased APC activation in the mucosa. Furthermore, this design elicited stronger T cell responses than bare RBD dimers, likely due to enhanced cross-presentation via Fc–FcRn binding^45^. These may collectively contribute to the induction of robust tissue-resident memory CD4/8^+^ T cell and Th17 CD4^+^ T cell responses, as well as elevated IgA titers in the respiratory tract by our framework or antigens. Since SARS-CoV-2 can invade the brain by infecting the olfactory bulb^46,47^ thereby causing brain lesions and neuropathy in both the acute and recovery phases^48^, robust mucosal immune protection from IgA and tissue-resident T cells appears to be particularly important. This further emphasizes the value of the framework reported in this study.

In contrast to the study by Zhu et al.^20^, where a T4 fibritin foldon was used to trimerize the SARS-CoV-2 S protein fused with an IgG1 Fc lacking C1q binding sites, our study removed the FcγR binding sites (L234A, L235A, G237A), which may minimize side effects, such as CRS induced by Fc–FcγR crosslinking^49^, while reducing antigen exhaustion caused by excessive FcγR binding. Unlike approaches using an IgG4 Fc with modifications to silence effector function by Zhang et al.^50^, our study introduced the YTE mutation into IgG1 Fc to enhance FcRn binding and further extend antigen half-life, and offering more application examples from mono-, bi-, tetra- and muti- valent antigens, presenting comprehensive advantages of employing artificial antibody strategy to solve vaccine problems. Thus, together with the previous researches, these findings strengthen the case for using the antibody scaffolds for antigen delivery to achieve broad-spectrum and enhanced immune effects, helping to advance the field.

It is speculated that antigens developed using the artificial antibody-inspired framework could mimic antibodies, bind B cells via relevant immune receptors, trigger crosslinking, and promote B cell receptor-mediated endocytosis. This would lead to increased B cell proliferation and a stronger germinal center response supported by follicular CD4^+^ helper T cells^51^; however, further evidence is required. This mechanism may partially explain the potent humoral immune response observed in this study despite a low antigen dose.

This framework facilitates the preparation of monovalent, bivalent, trivalent, and tetravalent antigens. While larger forms, such as hexavalent antigens, may be feasible, they would likely suffer from reduced yield and stability. For instance, bivalent and tetravalent antigen yields via ExpiCHO-S transient transfection followed by protein A-affinity chromatography and SEC purification were ~50 mg/L and 10 mg/L (data not shown), respectively, implying that higher valency lowers output and stability. Additionally, multivalent assembly may affect epitope exposure. Therefore, combining multiple bivalent or tetravalent antigens offers a practical approach for high efficacy and broad protection, as demonstrated by the pan-spectrum HCoV protection observed in the current study. Moreover, assembling the current IgG framework into a hexamer, as in IgM, remains a highly attractive solution^52^.

This study has certain limitations, and validation using models that more closely represent human respiratory anatomy and immune environments is necessary, as differences between rodents and humans could affect antigen response and immunity. Nevertheless, it is anticipated that immune protection will be improved in humans, given that human IgG1 Fc binds hFcRn with greater affinity than mFcRn. This hypothesis could be tested hACE2/hFcRn double knock-in mice. Although human clinical trials would provide the most relevant data, large-scale trails assessing broad-spectrum protection and mucosal immunity remain challenging due to sampling difficulties.

In summary, this study demonstrates that the application of a universal antigen design based on a modified IgG1 heavy chain yields safe, broad-spectrum, mucosal, and long-lasting protection. This approach presents a viable strategy for HCoV vaccine development. Furthermore, the study provides a comprehensive antigen design solution and introduces promising vaccine candidates targeting current and emerging HCoVs, with an emphasis on SARS-CoV-2 variants.

## Methods

### Cells and viruses

VeroE6 (American Type Culture Collection [ATCC], Manassas, VA, USA) cells were cultured in Dulbecco modified Eagle medium (DMEM, Gibco, Grand Island, NY, USA) supplemented with 10% fetal bovine serum (FBS, Gibco) at 37 °C and 5% CO_2_ for passage, titration, and neutralization of SARS-CoV-2. LLC-MK2 cells (ATCC) were cultured in DMEM supplemented with 10% FBS at 35 °C and 5% CO_2_ for passage, titration, and neutralization of HCoV NL63, according to standard protocols.

HACE2-stably overexpressing HEK293T cells, as well as hFcRn- or mFcRn-stably overexpressing MDCK cells, were established through lentiviral transduction. Lentiviral vectors, pCDH-CMV-hACE2, and pCDH-CMV-FCGRT (human or mouse)-P2A-B2M (human or mouse)-T2A-EGFP-EF1α-Puro, were purchased from MiaoLing Biotechnology (Wuhan, China). Lentiviruses were produced by co-transfecting HEK293T cells with the primary plasmids, pCG8.91, and pMD2.G, at a mass ratio of 2:3:1. After 48 h, the supernatants were combined with fresh medium and used to infect HEK293T or MDCK cells. Fluorescence intensity was measured 48 h post-infection with green fluorescent protein (GFP). Puromycin selection was applied at a final concentration of 2–4 μg/mL, adjusted based on cell sensitivity, until GFP-positive cells comprised more than 95% of the population.

All viruses were sourced from the National Virus Resource at the Wuhan Institute of Virology, Chinese Academy of Sciences: SARS-CoV-2 (PT: IVCAS6.7512; Beta: IVCAS6.7552; Delta: IVCAS6.7585; Omicron BA.1: IVCAS6.7600; Omicron BA.2: IVCAS 6.7617; Omicron BA.5: IVCAS 6.8981; Omicron XBB.1.9: IVCAS 6.9084; Omicron XBB.1.16: IVCAS6.9083; Omicron EG. 5: IVCAS6.9086; Omicron JN.1: IVCAS6.9355), and HCoV NL63 (IVCAS6.7565).

### Vaccine preparation

Codon-optimized RBD genes from SARS-CoV-2, including PT (GenBank: QHR63260.2), Beta (GenBank: UPE85823.1), Delta (GenBank: UWM25519.1), Lambda (GenBank: QSF03921.1), Omicron BA. 1 (GenBank: UVN39829.1), Omicron BA. 2.75 (GenBank: OQ300207.1), Omicron BA. 5 (GenBank accession number: OQ248383.1), Omicron XBB. 1.5 (GenBank: WEQ09113.1), Omicron XBB. 1.16 (GenBank: PP848050.1), and Omicron EG. 5 (GenBank: PP846672.1), as well as other HCoVs, namely OC43 (GenBank: AVR40344.1), NL63 (GenBank: APF29071.1), 229E (GenBank: APT69883.1) and HKU1 (GenBank: YP_173238.1), were cloned into the expression plasmid pXC17.4 (Lonza, Basel, Switzerland) constructed based on the framework of a modified human IgG1 heavy chain (L234A, L235A, G237A, M252Y, S254T and T256E).

ExpiCHO-S cells (Thermo Fisher Scientific, Waltham, MA, USA) were transiently transfected with the plasmids and cultured in a serum-free medium for 14 days. Supernatants were collected and purified using protein A-affinity chromatography (GenScript, Nanjing, China), followed by size-exclusion chromatography (SEC; Cytiva, Upsala, Sweden) to isolate the target proteins.

The recombinant proteins were analyzed by reduced or non-reduced sodium dodecyl sulfate-polyacrylamide gel electrophoresis (SDS–PAGE) or were directly used for subsequent studies. Each vaccine dose contained 50 µL with 1–5 µg of recombinant protein antigens, plus one adjuvant: either 20 µg of CF501 (MedChemExpress, Monmouth Junction, NJ, USA), 5 µg of Alu (Macklin, Shanghai, China), or 50 µL of AS03 (GSK, London, UK).

### Flow cytometric detection of binding capacity

Proteins were conjugated with Cy3 dye (MedChemExpress), incubated for 1 h at room temperature (~25 °C), and purified via ultrafiltration to remove unbound dye. The Cy3-labeled proteins were incubated with hACE2-HEK293T, hFcRn-MDCK, or mFcRn-MDCK cells in phosphate-buffered saline (PBS, pH = 7.2 or 6.4; Gibco) at 4 °C for 30 min, with WT HEK293T or MDCK cells as controls. Cells were washed thrice with PBS, fixed in 4% paraformaldehyde for 30 min, washed again, and analyzed by flow cytometry for surface fluorescence.

### Transwell-based transcytosis assays

Transcytosis assays were conducted using 0.4 μm Transwell filters (1.12 cm^2^) with collagen-coated polytetrafluoroethylene (Corning Costar, Corning, NY, USA). The filters were incubated overnight in complete medium and seeded with 1.0 × 10^5^ MDCK-hFcRn or MDCK-mFcRn cells per well. WT or hFcRn- or mFcRn-overexpressing MDCK cells were cultured for 4–6 days until reaching 100% confluence (transepithelial electrical resistance: 120–150 Ω × cm^2^; apparent permeability coefficient <5 × 10^−7 ^cm/s). Before experiments, cells were starved for 1 h in Hank’s Balanced Salt Solution (pH 6.0, adjusted with MES buffer; Gibco). Next, 200 nM protein was added to the apical Transwell chamber, and basilar samples were collected at 0, 1, 2, 4, and 8 h. Protein concentrations were measured by double-antibody sandwich enzyme-linked immunosorbent assay with mouse polyclonal capture antibodies (SinoBiological, Beijing, China) and mouse polyclonal detection antibodies (SinoBiological).

### Mucosal retention experiment

Proteins were labeled with Alexa Fluor (AF) 647 NHS ester dye (Thermo Fisher) in 0.1 M sodium bicarbonate buffer (pH 8.3) at 25 °C for 1 h, using a 1:5 molar ratio. The unbound dye was subsequently removed by ultrafiltration after the reaction. The labeled proteins (20 µg) were combined with CF501 adjuvant and administered i.n. to BALB/c mice (*n* = 3/group) after inhalational anesthesia with isoflurane. Vehicle mice received only the adjuvant. After intranasal delivery, mice were euthanized at 0, 3, or 6 h. Respiratory tissues were dissected, fixed, sectioned, and imaged for mucosal antigen retention analysis. Activated APCs were identified by staining with antibodies against CD80 and FcRn (CST, Boston, MA, USA), followed by colocalization analysis.

### Histopathology and immunofluorescence analysis

The brain, nasal turbinates, lungs, and trachea of mice or hamsters were dissected after natural death or euthanasia, and fixed in 4% paraformaldehyde for 7 days. Tissues were dehydrated and embedded in paraffin. Blocks were sectioned into 4 μm slices by a freezing microtome (Thermo Fisher Scientific) and stained with hematoxylin and eosin for histopathological analysis. After dehydration and sealing, slices were either stored or imaged using a microscopic imaging system (NIKON Digital Sight DS-FI2; Tokyo, Japan).

For tissue infection assessment, sections were blocked with 5% fat-free milk and incubated with rabbit monoclonal antibodies (CST) against the SARS-CoV-2 N protein. This was followed by incubation with a Cy3-conjugated secondary antibody (Bioqiandu, Wuhan, China) and DAPI (Sigma-Aldrich, St. Louis, MO, USA). Imaging was performed with a fluorescence microscope (Panoramic MIDI 3D; 3DHISTECH, Budapest, Hungary). Mean density (optical density/area) was measured with Image-Pro Plus 6.0 (Media Cybernetics, Rockville, MD, USA) from at least three panoramic views per section to assess viral infection.

### Animal vaccination and challenge

Animals were sourced from GemPharmatech Co., Ltd. (Nanjing, China), and housed in a specific pathogen-free facility. SARS-CoV-2 challenge experiments were performed in an animal biosafety level (ABSL)-3 laboratory, while the HCoV NL63 challenge experiment was conducted in an ABSL-2 laboratory.

Adult female BALB/c mice (*n* = 4–6), hACE2-transgenic C57BL/6 J mice (*n* = 5), and Syrian hamsters (*n* = 5) received two i.m. or i.n. vaccinations 3 weeks apart (relevant dosages, administration routes, and formulations were shown with the figures or legends for each experiment). Blood samples were collected from the ophthalmic vein 14 and 28 days after the first dose, and every 4 weeks thereafter up to 48 weeks. Blood was processed overnight at 4 °C, and centrifuged at 3000 rpm for 10 min to obtain sera.

Two weeks after vaccination, mice were transferred to ABSL-3 or ABSL-2 laboratories for SARS-CoV-2 (PT, Beta, Delta: 1 × 10^3^ plaque-forming units [PFUs]; BA.1, BA.2, BA.5, XBB.1.16: 1 × 10^4^ PFUs) or HCoV NL63 (1 × 10^5^ PFUs) i.m. or i.n. challenge, respectively. Meanwhile, hamsters were transferred to an ABSL-3 laboratory to receive SARS-CoV-2 (PT, XBB.1.16: 1 × 10^5^ PFUs) i.n. challenge. All animals underwent airway isoflurane anesthesia before challenge.

Body weight and survival were monitored throughout the two-week experimental period. At the experimental endpoint—either upon natural death or euthanasia—the brain, nasal turbinates, lungs, and trachea were harvested for viral load, histopathology, and immunofluorescence analyses.

### Enzyme-linked immunosorbent assay (ELISA) for specific antibody detection

Ninety-six-well polystyrene high-binding flat-bottom plates (Greiner, Ludwigsburg, Germany) were coated overnight at 4 °C with RBD or S1 proteins at 1 μg/mL in 100 μL/well. Plates were washed thrice with PBST (PBS containing 0.5% Tween-20) and then blocked with 5% nonfat dried milk at 37 °C for 1 h. Gradient-diluted serum or respiratory lavage samples were then added and incubated at room temperature for 2 h. After five additional washes, horseradish peroxidase-conjugated antibodies (Proteintech, Wuhan, China) were applied and incubated at 37 °C for 1 h. Next, 100 μL of tetramethyl benzidine solution (Proteintech, Wuhan, China) was added, incubated for 15 min, and then 50 μL of 2 M H_2_SO_4_ was added per well. Absorbance was measured at 450 nm using a Synergy H1 microplate reader (BioTek, Winooski, VT, USA). Results with values exceeding twice those of the control were considered positive.

### Flow cytometric analysis of cellular immune responses

Spleens from vaccinated BALB/c mice (*n* = 5) were dissected, and splenocytes were isolated and plated at 1 × 10^7^ cells/well in a 96-well U-bottom plate. Each well received 50 µL of stimulant and 5 µg/mL Brefeldin A (Absin, Shanghai, China), with PMA (BioLegend, San Diego, CA, USA) as the positive control and Roswell Park Memorial Institute (RPMI)-1640 (Gibco) as the negative control. Splenocytes were restimulated with RBD peptides (10 µg/mL; GenScript), incubated for 8 h, stained with Zombie Aqua dye (BioLegend) for 15 min, blocked with TruStain FcX™ (anti-mouse CD16/32; BioLegend) for 15 min, and incubated with cell typing antibodies (BioLegend; CD3e-APC/Cy7, CD4-FITC, CD8-PE/Cy7, CD44-APC, CD62L-BV421, CD69-PE/Dazzle 594 and CD103-PerCp/Cy5.5) diluted in PBS (0.5% BSA) for 30 min, each at 4 °C. After fixation with 100 µL of fixation buffer (BioLegend) and permeabilization with a staining permeabilization wash buffer (Biolegend), intracellular antibody staining was performed (BioLegend; IFN-γ-BV605, IL-4-BV711 and IL-17A-BV650) at 4 °C for 45 min before flow cytometric analysis. The data were analyzed by FlowJo software (version 10.2; Treestar, Ashland, OR).

### Neutralization test

VeroE6 cells (1.5 × 10^5^ cells/well) were seeded in 24-well plates and incubated overnight at 37 °C. Gradient-diluted sera in 200 μL FBS-free DMEM were incubated with 300 PFUs of SARS-CoV-2 or variants for 1 h at 37 °C. Subsequently, 150 μL of the mixture was added to each well in duplicate and incubated for 1 h at 37 °C. The mixture was then removed and replaced with 1 mL of 2% FBS-DMEM containing 0.9% methylcellulose. After 3 days, plates were fixed with 10% formaldehyde, stained with 0.1% crystal violet, and plaques were counted manually to determine the 50% plaque-reduction neutralization titer (PRNT_50_).

Alternatively, LLC-MK2 cells (5 × 10^5^ cells/well) were seeded in 96-well plates. Serum samples were serially diluted in DMEM containing 2% FBS, mixed with an equal volume of NL63 virus (containing 100 TCID₅₀), and incubated at 35 °C for 1 h. The mixture was added to LLC-MK2 cells and incubated for five days at 35 °C. Supernatants underwent RNA extraction and qPCR; viral copies were quantified for the PRNT_50_.

### Measurement of tissue viral load using the plaque formation method or quantitative reverse transcription polymerase chain reaction (qRT-PCR)

To measure the viral titer in tissues, VeroE6 cells were seeded into 24-well plates and cultured overnight to establish a confluent monolayer. Tissue samples (200 mg) from rodent brain, lungs, or turbinates were homogenized in 1 mL of serum-free medium, and 100 μL of the homogenate underwent a six-step, 10-fold serial dilution. Before infection, the supernatants were aspirated from the 24-well plates, and 150 μL of diluted homogenate was added to each well in duplicate. After a 1-h incubation, the inoculum was discarded, and cells were covered with 1 mL of DMEM containing 1% methylcellulose and 2% FBS. At 72 h post-infection, cells were fixed with 8% formaldehyde and stained with 5% crystal violet. Plaques were manually counted, and viral titers in the tissues were calculated.

To detect and quantify viral copies, tissue specimens from endpoint animals—including brain, lung, and nasal turbinate—were mechanically homogenized in DMEM. The total RNA was extracted using the RNeasy Mini Kit (Qiagen, Hilden, Germany) per the manufacturer’s instructions, then reverse-transcribed into cDNA using the HiScript II Reverse Transcriptase Kit (Vazyme, Nanjing, China). Viral RNA copies were quantified via qRT-PCR on the QuantStudio I instrument (Applied Biosystems, Carlsbad, CA, USA) using a standard curve method with primers targeting the S gene.

### Data analysis

Data were analyzed using Prism software (version 9.0; GraphPad, San Diego, CA, USA) and are presented as the mean ± standard error of the mean (SEM). Unpaired *t*-tests with the Mann–Whitney U-tests were employed to determine significance. *P* values < 0.05 were regarded as significant. The schematic diagrams were created on the MedPeer platform (https://image.medpeer.cn/show/index/home).

### Ethics statement

Animal experiments were performed in strict accordance with the guidelines of the Regulations on the Administration of Experimental Animals of China. Experimental protocols were approved by the Experimental Animal Care and Use Committee of the Wuhan Institute of Virology, Chinese Academy of Sciences (ethics nos.: WIVAF25202302 and WIVA25202313).

## Supplementary information

Supplementary information

## Data Availability

The data associated with this study are included in the main text and Supplementary Materials. Inquiries regarding materials, data, and the elaboration of methods are available from the corresponding author X.P. (http://panxy@wh.iov.cn).
