# Supplementary material for: Universal broad-spectrum mucosal vaccine design for human coronaviruses inspired by artificial antibodies
Source: NPJ Vaccines. 2026 Jan 28;11:55. doi: 10.1038/s41541-026-01375-2 (PMC12921044; doi:10.1038/s41541-026-01375-2)
Supplement: Supplementary file 1 — Supplementary information [file 41541_2026_1375_MOESM1_ESM.pdf]

Supplementary Materials for

Universal broad-spectrum mucosal vaccine design for human coronaviruses inspired by artificial antibodies

Yan Wu[1,5], Jia Lu[2,5], Lijuan Fang[3,5], Xinlan Chen[2], Chenshu Zhao[1], Zhongfa Zhang[4], Xuerui Zhu[1], Xiao Gao[2], Haoyu Li[2], Yingrui Yan[2], Jian Shi[3], Jing Zhang[3], Pengfei Zhou[3], Xiaoyan Pan[1,2✉]

[1]State Key Laboratory of Virology and Biosafety, Wuhan Institute of Virology, Chinese Academy of Sciences, Wuhan 430207, China

[2]University of the Chinese Academy of Sciences, Beijing 101400, China

[3]Wuhan YZY Biopharma Co., Ltd., Wuhan 430040, China

[4]Hubei University, Wuhan 430062, China

[5]These authors contributed equally

✉ Email: panxy@wh.iov

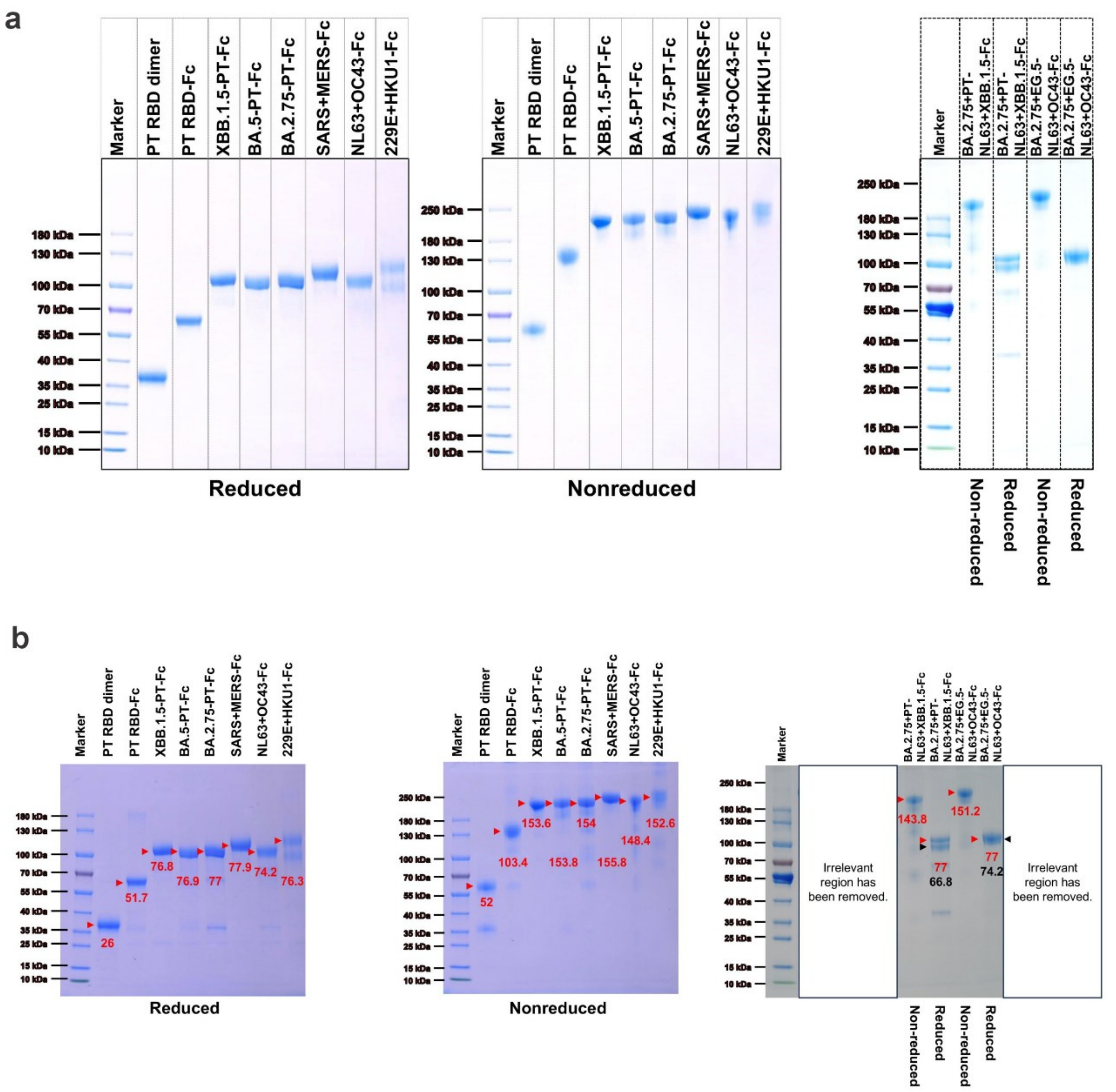

Figure S1 Antigen detection by reduced and non-reduced SDS-PAGE.
Electrophoretic analysis of the antigens (2 µg each) after Coomassie light blue staining. Theoretical molecular weights (kDa) were annotated on unprocessed gel images (**b**), with irrelevant areas removed. Gel images subjected to processing, such as contrast enhancement and brightness adjustment, were displayed as (**a**). Owing to glycosylation modifications, actual molecular weights exceeded the theoretical values.

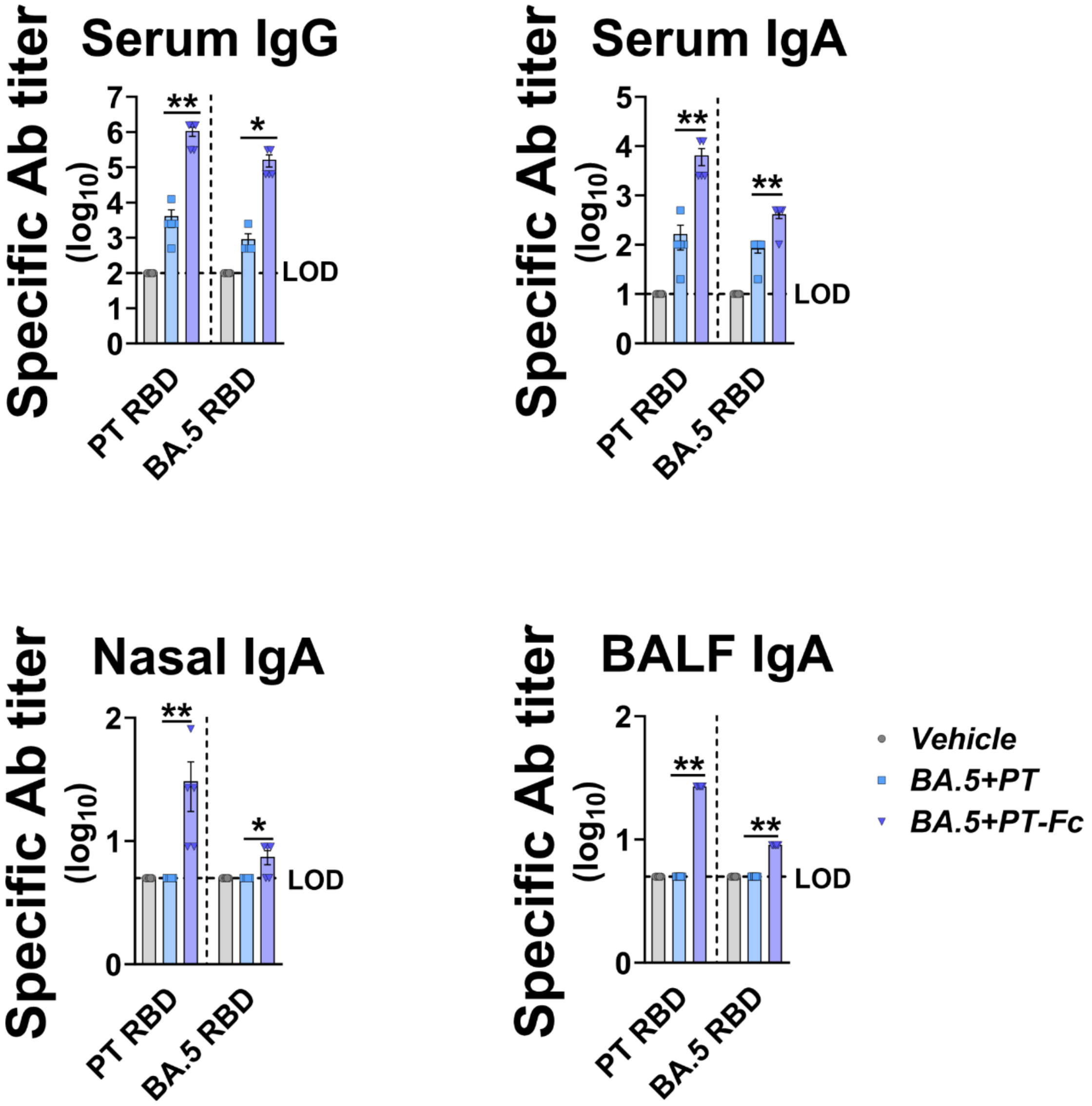

Figure S2 Fc triggers a robust antibody response to RBDs. Female BALB/c mice (*n* = 5) were vaccinated with equal molar mass (~0.008 nM) of bare BA.5+PT (~0.4 µg) and BA.5+PT -Fc (~0.6 µg) antigens, according to the same scheme as XBB.1.5+PT-Fc; two weeks post-vaccination, specific antibody titers were detected in sera, nasal and bronchoalveolar lavage fluid samples using ELISA. The limit of detection (LOD) was established as follows: 100 for serum IgG, 10 for serum IgA, 5 for nasal and BALF IgA. Unpaired t-tests with Mann–Whitney U-tests were employed, $*p < 0.05$, $**p < 0.01$.

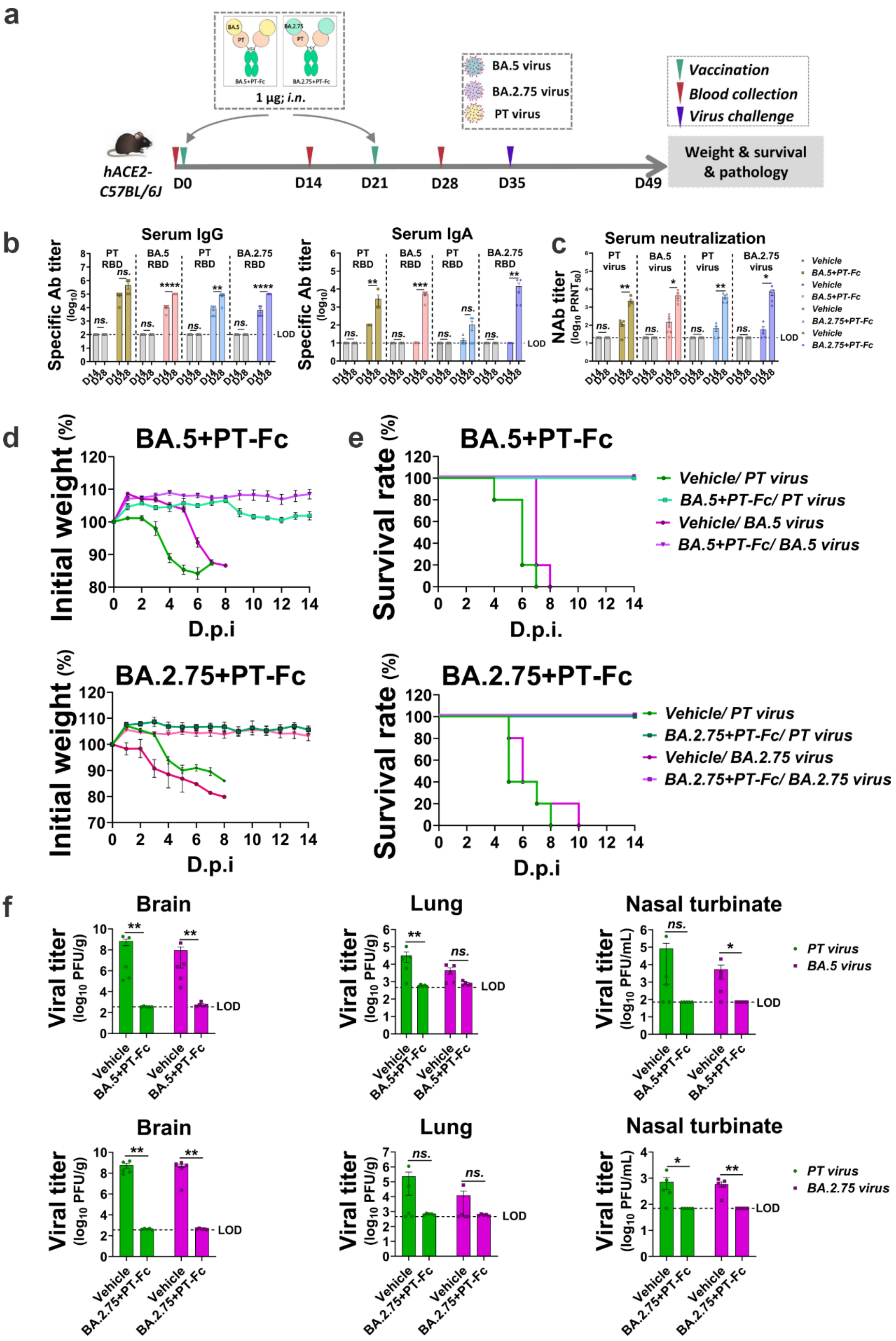

Figure S3 Two bivalent antigens achieve complete cross-protection in a lethal challenge model. (**a**) Immunization and challenge experimental scheme. Adult hACE2-trangenic C57BL6/J mice (*n* = 5) were intranasally vaccinated with 1 μg of BA.5+PT-Fc or BA.2.75+PT-Fc adjuvanted with CF501. Mice were vaccinated using a

two-dose scheme, and serum samples were collected every two weeks for antibody detection. A lethal dose of SARS-CoV-2 (PT, BA.5, and BA.2) was administered intranasally two weeks after the second dose. Body weight and survival were monitored for two weeks, and brain, lung, and turbinate tissues were collected from the endpoint animals for viral load detection and pathological examination. (**b**) Sera IgG and IgA cross-binding detection against RBDs (PT, BA.5, and BA.2.75) after the first and second doses. The limit of detection (LOD) for serum IgG and IgA was 100 and 10, respectively. (**c**) $PRNT_{50}$ tested against authentic SARS-CoV-2 variants, including PT, BA.5, and BA.2. The LOD was 20. (**d**, **e**) Body weight (d) and survival (e), with vehicle groups as controls. (**F**) Viral loads in the brain, lungs, and turbinates at the experimental endpoint, the LOD for brain, lung, and turbinate was 350, 466 and 70 PUF/g, respectively. Unpaired t-tests with Mann–Whitney U-tests were employed, ns: $p > 0.05$, $^{*}p < 0.05$, $^{**}p < 0.01$, $^{***}p < 0.001$, $^{****}p < 0.0001$.
